# Measles Outbreak Associated with a Migrant Shelter — Chicago, Illinois, February–May 2024

**DOI:** 10.15585/mmwr.mm7319a1

**Published:** 2024-05-16

**Authors:** Kimberly Gressick, Amy Nham, Thomas D. Filardo, Kendall Anderson, Stephanie R. Black, Katherine Boss, Maribel Chavez-Torres, Shelby Daniel-Wayman, Peter Dejonge, Emily Faherty, Michelle Funk, Janna Kerins, Do Young Kim, Alyse Kittner, Colin Korban, Massimo Pacilli, Anne Schultz, Alexander Sloboda, Shane Zelencik, Arti Barnes, Joshua J. Geltz, Jodi Morgan, Kyran Quinlan, Heather Reid, Kevin Chatham-Stephens, Tatiana M. Lanzieri, Jessica Leung, Chelsea S. Lutz, Ponesai Nyika, Kelley Raines, Sumathi Ramachandran, Maria I. Rivera, Jordan Singleton, Dennis Wang, Paul A. Rota, David Sugerman, Stephanie Gretsch, Brian F. Borah, Chicago Department of Public Health Measles Response Team, Chicago Department of Public Health Measles Response Team, Ashley Becht, Danielle Belanger, Marco Ciaccio, Anna Esquivel, Molly Gabaldo, Kevin Hansen, David Juen, Gira Patel, Bethlehem Solomon, Karrie-Ann Toews, Christy Zelinski

**Affiliations:** ^1^Epidemic Intelligence Service, CDC; ^2^Chicago Department of Public Health, Chicago, Illinois; ^3^Division of Viral Diseases, National Center for Immunization and Respiratory Diseases, CDC; ^4^Career Epidemiology Field Officer Program, CDC; ^5^Illinois Department of Public Health; ^6^Immunization Services Division, National Center for Immunization and Respiratory Diseases, CDC; ^7^Coronavirus and Other Respiratory Viruses Division, National Center for Immunization and Respiratory Diseases, CDC.; Chicago Department of Public Health; Chicago Department of Public Health; Chicago Department of Public Health; Chicago Department of Public Health; Chicago Department of Public Health; Chicago Department of Public Health; Chicago Department of Public Health; Chicago Department of Public Health; Chicago Department of Public Health; Chicago Department of Public Health; Chicago Department of Public Health.

**Summary**

**What is already known about this topic?**

Measles, a highly contagious respiratory virus, was declared eliminated from the United States in 2000; however, with ongoing global transmission, infections in the United States still occur. Receipt of 1 and 2 doses of measles vaccine is 93% and 97% effective, respectively, in preventing measles.

**What is added by this report?**

Fifty-seven measles cases were associated with residence in or contact with persons in a migrant shelter in Chicago, Illinois. Most cases occurred in unvaccinated persons. A prompt and coordinated response with a high-coverage mass vaccination campaign reduced the size and duration of the outbreak.

**What are the implications for public health practice?**

Ensuring high measles vaccination coverage during an outbreak can control measles spread and prevent wider transmission.

## Abstract

Measles, a highly contagious respiratory virus with the potential to cause severe complications, hospitalization, and death, was declared eliminated from the United States in 2000; however, with ongoing global transmission, infections in the United States still occur. On March 7, 2024, the Chicago Department of Public Health (CDPH) confirmed a case of measles in a male aged 1 year residing in a temporary shelter for migrants in Chicago. Given the congregate nature of the setting, high transmissibility of measles, and low measles vaccination coverage among shelter residents, measles virus had the potential to spread rapidly among approximately 2,100 presumed exposed shelter residents. CDPH immediately instituted outbreak investigation and response activities in collaboration with state and local health departments, health care facilities, city agencies, and shelters. On March 8, CDPH implemented active case-finding and coordinated a mass vaccination campaign at the affected shelter (shelter A), including vaccinating 882 residents and verifying previous vaccination for 784 residents over 3 days. These activities resulted in 93% measles vaccination coverage (defined as receipt of ≥1 recorded measles vaccine dose) by March 11. By May 13, a total of 57 confirmed measles cases associated with residing in or having contact with persons from shelter A had been reported. Most cases (41; 72%) were among persons who did not have documentation of measles vaccination and were considered unvaccinated. In addition, 16 cases of measles occurred among persons who had received ≥1 measles vaccine dose ≥21 days before first known exposure. This outbreak underscores the need to ensure high vaccination coverage among communities residing in congregate settings.

## Investigation and Results

Measles is a highly contagious respiratory virus with the potential to cause severe complications, hospitalization, and death (*1*). Although measles was declared eliminated from the United States in 2000, global transmission is ongoing (*2*). Receipt of 1 and 2 doses of measles vaccine is 93% and 97% effective, respectively, in preventing measles (*1*). 

Since August 2022, approximately 41,000 migrants have arrived in Chicago, Illinois from the U.S. southern border (*3*); most (88%) are from Venezuela, a country with a recent decline in routine childhood immunization coverage, including with measles vaccine (*4*). On February 22, 2024, approximately 12,000 persons were residing in 27 temporary migrant shelters operated by the city of Chicago. The largest shelter (shelter A) is a congregate setting with shared sleeping areas, dining area, and bathrooms. On February 22, 2024, approximately 2,100 persons resided in shelter A, with some rooms housing 500 or more persons.

### Index Patient

A male shelter A resident aged 1 year developed a rash on February 26, 2024, and was hospitalized on February 27 with suspected measles. On March 4, when the Chicago Department of Public Health (CDPH) was first notified of the suspected case, confirmatory measles testing with real-time reverse transcription–polymerase chain reaction (RT-PCR) was requested by CDPH. The child had arrived in the United States >5 months earlier and had received 1 dose of measles, mumps, and rubella (MMR) vaccine 5 weeks before rash onset^†^; he had no recent travel or known exposure to measles. Upon confirmation of wild-type measles infection by measles vaccine assay (MeVA)^§^ on March 7, CDPH alerted residents and staff members the same evening and arranged a vaccination event for the next morning. Given the highly congregate nature of shelter A, CDPH considered anyone who had been inside the shelter during February 22–27, the index patient’s infectious period at shelter A, to be exposed.

### Case Identification

A shelter A–associated case was defined as an RT-PCR–confirmed, wild-type measles infection in a person with a shelter A measles exposure, either by virtue of residing in, working at, or having a known epidemiologic link to persons from shelter A with a confirmed measles infection, during February 26–May 13. Laboratory confirmation included measles RT-PCR testing at the Illinois Department of Public Health Laboratory. To distinguish measles vaccine reaction from wild-type measles infection, laboratory confirmation required MeVA testing be performed by the Minnesota Department of Health Public Health Laboratory for persons who had received measles vaccine 5–21 days before rash onset. Among exposed persons who did not have a rash (but who had measles signs and symptoms, such as fever, cough, coryza, or conjunctivitis), RT-PCR collection date was used to determine the need for MeVA testing. For all cases, standard genotyping was attempted for available specimens. This activity was reviewed by CDC, deemed not research, and was conducted consistent with applicable federal law and CDC policy.^¶^

### Additional Cases

During February 26–May 13, CDPH confirmed 57 shelter A–associated measles cases, including 52 among residents, three among staff members, and two among community members (Figure) (Table). The median age of persons with confirmed infections was 3 years (range = 0–52 years); most were originally from Venezuela (43; 84%) and arrived in the United States a median of 124 days (range = 56–202 days) before rash onset. Most persons (41; 72%) did not have documentation of measles vaccination and were considered unvaccinated. Among all cases, 16 (28%) occurred among persons who had documentation of ≥1 measles vaccine dose ≥21 days before first known exposure, and four (7%) occurred among persons who had documentation of ≥2 measles vaccine doses. The median age of previously vaccinated persons with confirmed measles infections was 9.5 years (range = 1–49 years); seven patients (44%) were aged <5 years. Two cases occurred among persons who resided at shelter A during February 22–March 7, but had resettled or transferred to less crowded shelters with private sleeping areas after March 7; no secondary cases occurred at those shelters. As of May 13, identical measles genotype D8 sequences were identified from 52 case specimens; the remaining five isolates could not be sequenced. Fifty-one persons (89%) were hospitalized for either or both isolation and measles complications; no deaths were reported. As of May 13, the date of last known exposure at shelter A was April 5. 

**FIGURE:**
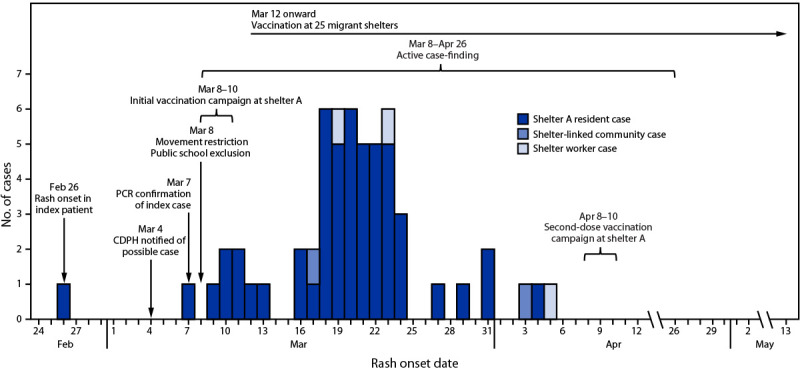
Measles cases associated with a migrant shelter (shelter A),* by rash onset date^†^ and public health interventions^§^ — Chicago, Illinois, February 26–May 13, 2024 **Abbreviations:** CDPH = Chicago Department of Public Health; PCR = polymerase chain reaction. * Shelter A resident cases were defined as those among persons exposed while residing at Shelter A. Shelter-linked community cases were defined as those among persons exposed outside of shelter A and epidemiologically linked to a case in a shelter A resident. Shelter worker cases were defined as those among persons exposed while working at shelter A. ^†^ Two persons with unknown or no rash onset were included by symptom onset date. ^§^ Interventions included active case-finding, vaccination events, and movement restriction.

**TABLE:** Characteristics* of persons with confirmed measles infections associated with a migrant shelter (N = 57) — Chicago, Illinois, February 26–May 13, 2024

Characteristic	No. (%)
**Sex**	
Female	30 (53)
Male	27 (47)
**Age group**	
<6 mos	4 (7)
6 mos–4 yrs	29 (51)
5–19 yrs	6 (11)
20–49 yrs	17 (30)
≥50 yrs	1 (2)
**No. of verified measles vaccine doses received^†^**	
1	12 (21)
≥2	4 (7)
None or unknown	41 (72)
**Country of origin^§^**	
Venezuela	43 (84)
Peru	4 (8)
Ecuador	2 (4)
Chile	1 (2)
Unknown	1 (2)
**Shelter resident status**	
Shelter A resident	52 (91)
Shelter worker	3 (5)
Shelter-linked community member	2 (4)

* Race data were reported as other or unknown for 25 (44%) persons; all were of Hispanic or Latino ethnicity.

^†^ Occurred ≥21 days before rash onset.

^§^ Documented as the country of last residence before beginning migration. One U.S.-born shelter resident and five nonshelter residents were excluded.

## Public Health Response

After identification of the index case, CDPH instituted a mass campaign to provide vaccination and verify vaccination records, active case-finding, and shelter movement restrictions (Figure). To deliver culturally and linguistically accessible messaging about measles infection and the importance of vaccination and quarantine, CDPH collaborated with trusted community health workers and leaders. These persons, who included “promotores de salud,” were effective liaisons for communicating messages from CDPH because they were fluent in Spanish and possessed insight and understanding of the community served.

### Vaccination

CDPH implemented a rapid and comprehensive vaccination campaign at shelter A during March 8–10, within 1 day of confirmation of the index case (Figure). Staff members verified physical vaccination records and vaccination status in Illinois’ immunization information system. All nonpregnant residents aged ≥6 months without documentation of previous measles vaccination and residents aged ≥1 year who had received a first dose ≥28 days earlier were offered MMR vaccination.** Shelter staff members and community partners were engaged to communicate the importance of vaccination and were recommended to provide evidence of measles immunity themselves.

During March 8–10, records documenting previous measles vaccination were verified for 784 (44%) of the 1,801 residents, and 882 (49%) eligible residents received MMR vaccine. By March 11, a total of 1,666 (93%) of 1,801 residents at shelter A had documentation of receipt of ≥1 dose of measles vaccine. As of May 13, CDPH had led approximately 130 mass vaccination events across 25 Chicago migrant shelters and administered approximately 9,500 MMR vaccine doses, prioritizing the shelters that had previously received residents from shelter A (all of whom were presumed to be exposed) and shelters with pregnant women and young children. This strategy included additional vaccination events at shelter A beginning on March 25, with a focused second-dose vaccination campaign during April 8–10 (Figure). 

### Active Case-Finding

CDPH began active case-finding at shelter A on March 8 (Figure). Medical and shelter staff members walked bed to bed to screen residents for measles signs and symptoms.^††^ On the basis of the degree of clinical suspicion for measles,^§§^ symptomatic residents were either tested and remained on site or were immediately transported to a hospital for testing and isolation. The median interval from rash onset to isolation was 1 day, ranging from 3 days before to 3 days after rash onset.

### Movement Restriction

All shelter A residents without evidence of receipt of ≥1 dose of measles vaccine ≥21 days before first known exposure were advised to quarantine in shelter A until 21 days from first MMR vaccination or, if unvaccinated, 21 days from last known exposure at shelter A. School exclusion for children in quarantine began on March 8 (Figure). Quarantine remained voluntary rather than imposed by city officials or law enforcement to encourage continued cooperation between CDPH and shelter A residents. Twenty-two family units with members who were at highest risk for infection (i.e., infants aged ≤6 months, nonimmune pregnant women, and immunocompromised persons) were transferred during March 11–12 to a repurposed hotel for quarantine. Intake of new residents to shelter A was halted on March 8, and movement of shelter A residents to nonquarantine shelters only occurred for persons with documentation of receipt of ≥1 dose of measles vaccine. Because of the inability to isolate symptomatic persons within shelter A, residents with laboratory-confirmed measles or high clinical suspicion of measles were isolated in Chicago hospitals for the remainder of their infectious periods.

## Discussion

After identification of a large measles outbreak among migrants, primarily from Venezuela, who resided in a shelter, active case-finding and rapid mass vaccination of residents likely reduced transmission and outbreak size and duration (*5*). Engagement of community partners contributed to the success of these public health interventions. 

Measles is a highly contagious respiratory virus (*1*), and this outbreak occurred in a densely populated congregate setting with high potential for transmission. Isolation space was needed although lacking for public health control measures, both at shelter A and in the larger community, placing a strain on Chicago hospitals. Persons isolated in hospitals occupied airborne infection isolation rooms during their infectious period or until measles was ruled out, underscoring the importance of having dedicated isolation space outside of hospitals for patients without medical need.

Measles is preventable with a highly effective vaccine (*1*); however, the national first-dose measles vaccination coverage among Venezuelan residents aged ≥12 months declined from 96% to 68% during 2017–2021 (*4*). Decreases in measles vaccination coverage, attributed to the disruption of routine immunization services during the COVID-19 pandemic, have been observed worldwide (*6*).

Measles postexposure prophylaxis (PEP) with MMR vaccine must be administered within 72 hours of exposure to be effective in preventing measles^¶¶^ (*1*). Mass vaccination at shelter A occurred outside the window for PEP after the initial exposure but likely prevented measles cases resulting from later exposures, thereby limiting the size and duration of the outbreak (*5*). Residents’ ineligibility for PEP because of the 6-day delay in notification to CDPH highlights the importance of prompt notification of suspected and confirmed measles cases to health departments.

The percentage of measles cases among persons with a history of previous vaccination was higher than that reported through recent national surveillance in the United States (*7*), likely owing to a high degree of exposure from the congregate living situation (*5*). Infections in vaccinated persons can occur because of primary vaccine failure, in which an immunologic response to vaccination does not occur, or secondary vaccine failure, in which infection occurs despite previous response to vaccination. Primary vaccine failure occurs in approximately 4% of recipients of 1 MMR dose and is rare among recipients of 2 MMR doses (*6*). Secondary vaccine failure generally occurs because of prolonged or close exposure to measles virus and has been observed in congregate settings (*8**,**9*). A full assessment of whether these infections are due to primary or secondary vaccine failure is ongoing.

Measles was declared eliminated from the United States in 2000; however, with ongoing global transmission, infections in the United States still occur (*2*). Although persons in the community affected by this outbreak had recently arrived in the United States, the index patient’s arrival in Chicago months before illness onset suggests that the disease was acquired locally. To date, a direct epidemiologic link to another case has not been identified. The risk for transmission within and outside of shelters can be mitigated by maintaining high MMR vaccination coverage among both established and newly arrived residents.

## Footnotes

* These authors contributed equally to this report.

† Up to 5% of persons who receive MMR vaccine will develop a vaccine reaction with rash, which is clinically indistinguishable from a rash resulting from wild-type measles infection.

§ After measles vaccination, vaccine strain measles virus can be detected with conventional measles RT-PCR, and MeVA is used to differentiate between vaccine strain and wild-type measles virus. The MeVA test is a real-time RT-PCR assay that detects only measles vaccine strains.

¶ 45 C.F.R. part 46.102(l)(2), 21 C.F.R. part 56; 42 U.S.C. Sect. 241(d); 5 U.S.C. Sect. 552a; 44 U.S.C. Sect. 3501 et seq.

**
https://www.cdc.gov/vaccines/vpd/mmr/hcp/recommendations.html

†† Symptoms concerning for measles included fever, rash, cough, coryza, or conjunctivitis.

§§ High clinical suspicion included persons with fever and rash or fever and either cough, coryza, or conjunctivitis.

¶¶ Measles incubation period ranges from 11 to 12 days, with rash onset typically occurring 14 days after exposure.
